# *Staphylococcus aureus* PSM Peptides Modulate Human Monocyte-Derived Dendritic Cells to Prime Regulatory T Cells

**DOI:** 10.3389/fimmu.2018.02603

**Published:** 2018-11-13

**Authors:** Jennifer R. Richardson, Nicole S. Armbruster, Manina Günter, Jörg Henes, Stella E. Autenrieth

**Affiliations:** Department of Internal Medicine II, University of Tübingen, Tübingen, Germany

**Keywords:** monocyte-derived dendritic cells, *Staphylococcus aureus*, phenol-soluble modulins, immune tolerance, immunity, regulatory T cells

## Abstract

*Staphylococcus aureus* (Sa), as one of the major human pathogens, has very effective strategies to subvert the human immune system. Virulence of the emerging community-associated methicillin-resistant Sa (CA-MRSA) depends on the secretion of phenol-soluble modulin (PSM) peptide toxins e.g., by binding to and modulation of innate immune cells. Previously, by using mouse bone marrow-derived dendritic cells we demonstrated that PSMs in combination with various Toll-like receptor (TLR) ligands induce a tolerogenic DC phenotype (tDC) characterized by the production of IL-10 and impaired secretion of pro-inflammatory cytokines. Consequently, PSM-induced tDCs favored priming of CD4^+^CD25^+^FoxP3^+^ T_regs_ with suppressor function while impairing the Th1 response. However, the relevance of these findings for the human system remained elusive. Here, we analyzed the impact of PSMα3 on the maturation, cytokine production, antigen uptake, and T cell stimulatory capacity of human monocyte-derived DCs (moDCs) treated simultaneously with either LPS (TLR4 ligand) or Sa cell lysate (TLR2 ligand). Herein, we demonstrate that PSMs indeed modulate human moDCs upon treatment with TLR2/4 ligands via multiple mechanisms, such as transient pore formation, impaired DC maturation, inhibited pro- and anti-inflammatory cytokine secretion, as well as reduced antigen uptake. As a result, the adaptive immune response was altered shown by an increased differentiation of naïve and even CD4^+^ T cells from patients with Th1/Th17-induced diseases (spondyloarthritis and rheumatoid arthritis) into CD4^+^CD127^−^CD25^hi^CD45RA^−^FoxP3^hi^ regulatory T cells (T_regs_) with suppressor function. This T_reg_ induction was mediated most predominantly by direct DC-T-cell interaction. Thus, PSMs from highly virulent Sa strains affect DC functions not only in the mouse, but also in the human system, thereby modulating the adaptive immune response and probably increasing the tolerance toward the bacteria. Moreover, PSMα3 might be a novel peptide for tolerogenic DC induction that may be used for DC vaccination strategies.

## Introduction

Dendritic cells (DCs) are specialized antigen presenting cells (APCs) able to prime naïve T cells thereby inducing a primary immune response and maintaining self-tolerance (1). Initially DCs occur in an immature state, specialized for antigen uptake with a high endocytic capability (2). The recognition of pathogens via germ-line encoded pattern-recognition receptors, like Toll-like Receptors (TLRs), leads to DC maturation (3). This event is characterized by the loss of their endocytic capacities and the upregulation of CCR7, co-stimulatory molecules, and HLA-DR, necessary for homing into the draining lymph node and T-cell priming (4). Furthermore, DCs secrete pro-inflammatory cytokines, such as TNF-α, IL-6 or IL-12, which recruit other immune cells for pathogen clearance and contribute to T helper cell (Th) differentiation (5). Apart from inducing an efficient immune response, DCs are also crucial for maintaining immune tolerance in the steady-state. Although the specific phenotype of so-called tolerogenic DCs (tDCs) and the molecular mechanism involved in tolerance induction by these cells are not entirely defined (6–9) they are characterized by an immature phenotype and produce high amounts of anti-inflammatory cytokines, e.g., IL-10 and TGF-ß, which possess critical immunoregulatory functions like controlling/regulating the production of pro-inflammatory cytokines. They have the potential to induce regulatory T cell (T_reg_) expansion thereby impairing effector T cell responses (8, 10–12).

Various pathogens and tumors can induce tDCs and subsequent T_reg_ differentiation as immune escape strategy to impair clearance. This process is mediated by pathogenic products from e.g., *C. albicans, S. mansoni* and *V. cholerae*, which are partially used for the production of immunosuppressive drugs. These are widely used for therapy of autoimmune diseases or transplant rejections even though they have severe side effects by suppressing the entire host immune system (11, 13). Therefore, DC vaccination strategies by applying tDCs are an attractive alternative (8, 9, 13). Several clinical trials started to analyze the effect of tDCs as treatment option for patients with autoimmune disorders (8).

Phenol-soluble modulins (PSMs) are short amphipathic α-helical peptides, which are produced by highly virulent Staphylococci, such as community-associated Methicillin-resistant *Staphylococcus aureus* (Sa) promoting, e.g., cell lysis thereby evading clearance by immune cells (14, 15). Two types of PSMs are distinguished according to their length: α-type PSMs (~20–25 AA) and β-type PSMs (~44 AA) (16). The PSMα peptides are the most potent PSMs regarding cytolysis and highly contribute to the virulence of Sa (16, 17). Own previous studies with mouse bone-marrow derived DCs (BM-DCs) showed that PSMα3 prime tDCs when co-incubated with various TLR ligands (TLRL), regardless which TLR was activated. Molecularly, this event is characterized by the increased activation of the p38-CREB pathway, which in consequence leads to diminished pro-inflammatory cytokine production but increased IL-10 secretion. These PSM-induced tDCs favored priming of CD4^+^CD25^+^FoxP3^+^ T_regs_ with suppressor function (10, 12, 18). Thus, we hypothesized that PSMs of Sa likewise induce tDCs in the human system.

Herein, we show that PSMα3 penetrates and modulates human monocyte-derived DCs (moDCs) by altering the TLR2- or TLR4-induced maturation, inhibiting pro- and anti-inflammatory cytokine production and reducing antigen uptake, but producing indolamin-2,3-dioxygenase (IDO). As a result, the frequency of CD4^+^CD127^−^CD25^hi^CD45RA^−^Foxp3^hi^ T_regs_ is increased, while Th1 responses are diminished. Moreover, PSMα3-induced tDCs from healthy donors even enhanced differentiation of CD4^+^ T cells from patients with Th17-associated autoimmune diseases to T_regs_. Thus, PSMα3 might be a novel peptide for manipulating DCs to become tolerogenic for DC vaccination strategies.

## Materials and methods

### Research subjects

Buffy coats from healthy volunteers were obtained from the ZKT Tübingen GmbH. Fresh blood was obtained from healthy volunteers with informed consent. This was approved by the ethical review committee of the medical faculty of the Eberhard-Karls-University of Tübingen with the project number 633/2012BO2. Blood from patients with TH17-associated autoimmune diseases were obtained from the division of Rheumatology, Department of Internal Medicine II, University Hospital Tübingen. This was approved by the ethical review committee of the medical faculty of the Eberhard-Karls-University of Tübingen with the project number 046/2015BO2.

### Reagents

Formylated PSM peptides (PSMα3, δ-Toxin) were synthesized at the Interfaculty Institute of Cell Biology, Department of Immunology, University of Tübingen. FITC-labeled PSMα2 was synthesized at the Group of Hubert Kalbacher, Interfaculty Institute of Biochemistry, University of Tübingen. Sa cell lysate (Sa lysate) containing lipopeptides and specifically activating TLR2 was prepared from a protein A-deficient Sa mutant strain (SA113) and provided by Andreas Peschel, Interfaculty Institute of Microbiology and Infection Medicine, University of Tübingen.

### Isolation of peripheral blood mononuclear cells

Buffy coats or fresh blood was diluted with Dulbecco's PBS (Life Technologies) (Buffy Coats 1:7 blood: PBS; Fresh blood 1:1 blood: PBS). Peripheral blood mononuclear cells (PBMCs) were obtained by density gradient centrifugation at 2000 rpm for 20 min at room temperature with 35 mL cell suspension stacked on 15 mL Biocoll separation solution (Biochrom). The interphase containing the PBMCs was abstracted and washed twice with PBS. PBMCs were further used to generate human moDCs and for the isolation of naïve CD4^+^ T cells and CD4^+^ T cells.

### Generation of human monocyte-derived DCs

PBMCs were plated in a tissue-treated 6-well plate (6 × 10^6^ cells per well) in DC medium [RPMI1640 (Merck), 10% FBS (Sigma), 2 mM L-Glutamine (Life Technologies), 100 U/mL Penicillin-Streptomycin (Life Technologies), 1 × non-essential amino acids (Merck), 1 mM sodium pyruvate (Merck) and 50 μM 2-mercaptoethanol (Roth)] and incubated for 1 h at 37°C, 5% CO_2_. After that, wells were washed with medium and PBS discarding the non-adherent cells. 3 mL/well DC medium containing 50 ng IL-4 and 100 ng GM-CSF (both from Miltenyi) was added to the remaining cells. Cells were incubated for 6 d at 37°C, 5% CO_2_. Cytokines were again added on day 2 and day 4. At day 6 the cells were used for the following experiments. The purity of the moDC culture was always >90% of leukocytes (Figure S1).

### Cytokine/indolamin-2,3-dioxygenase production by moDCs

MoDCs (2.5 × 10^5^) were seeded in a 96-well plate and treated with 3 μg/mL Sa lysate or 100 ng/mL LPS in combination with or without PSMα3 (10 μM). For some experiments moDCs were treated with different concentrations of PSMα3 or additionally stimulated with 100 ng/mL Pam2CSK4 (for TLR2/TLR6; InvivoGen), 1 μg/mL Pam3CSK4 (for TLR1/TLR2; InvivoGen), 1 μg/mL CpG ODN 2395 (for TLR9; InvivoGen), 5 μg/mL Imiquimod (for TLR7; InvivoGen), 2 μg/mL Flagellin (for TLR5; InvivoGen) or 10 μg/mL LTA (for TLR2/TLR4; InvivoGen) in combination with or without PSMα3 (10 μM). Supernatants were collected after 6 h, 24 h or 48 h and analyzed for TNF, IL-10, IL-12 and IDO production, respectively. Cytokines and IDO in the supernatants were determined by sandwich ELISA [eBioscience (TNF, IL-10, IL-12), R&D Systems (IDO)] according to the manufacturer's instructions.

### Flow cytometry

For moDC surface marker analysis of the costimulatory and inhibitory molecules, moDCs (2 × 10^5^) were seeded in a 96-well plate and stimulated with 3 μg/mL Sa lysate or 100 ng/mL LPS with or without PSMα3 (10 μM) or PSMα3 alone for 6 h or 24 h. Cells were removed from the plate using Accutase (Sigma-Aldrich) and treated with IgG from human serum (1 μg of human IgG per 100,000 cells; Sigma-Aldrich) for 20 min at room temperature to avoid unspecific binding via Fc receptors. Cells were stained with ZombieAqua (BioLegend) to exclude dead cells and fluorochrome conjugated extracellular antibodies: HLA-DR BV650 (L243, BioLegend), CD11b BV510 (ICRF44, BioLegend), CD11c APC (MJ4-27G121, Miltenyi), CD11c PE-Cy7 (Bu15, BioLegend), CD40 FITC (5C3, eBioscience), CD80 PE-Cy7 (2D10, BioLegend), CD83 PE-Dazzle 594 (HB15e, BioLegend), CD86 BV605 (IT2.2, BioLegend), PD-L1 PE (29E.2A3, BioLegend), PD-L2 PE (MIH18), ILT3 PE (ZM4.1, BioLegend) for 20 min at 4°C. FACS buffer [PBS containing 1% FBS, 2 mM EDTA (Merck) and 0.09% NaN_3_ (Sigma-Aldrich)] was used for all incubations and washing steps. At least 50,000 cells were acquired using a LSR Fortessa flow cytometer (BD Biosciences) with the DIVA software (BD Biosciences) and were further analyzed using FlowJo 10.4.2 software (Tree Star).

### Phosphoflow

For the experiments analyzing phosphorylation of signaling cascades, moDCs (2 × 10^5^) were seeded in a 96-well plate and treated for 1 h with 100 ng/mL LPS with or without PSMα3 (10 μM) or PSMα3 alone. Cells were removed from the plate using Accutase and treated with IgG from human serum (1 μg of human IgG per 100,000 cells) for 20 min at room temperature to avoid unspecific binding via Fc receptors. Cells were stained with ZombieAqua (BioLegend) to exclude dead cells and fluorochrome conjugated extracellular antibodies: HLA-DR PE (L243; BD Biosciences) and CD11c APC/Cy7 (Bu15; BioLegend) for 20 min at 4°C. To detect intracellular p-p38 and p-NF-κB cells were fixed with 2% paraformaldehyde (VWR) in PBS, permeabilized with 90% freezing methanol (Applichem) overnight and stained with the primary Abs to phospho-p38 MAPK (Thr180/Tyr182; clone 12F8) or phospho-NF-κB p65 (Ser536; clone: 93H1) (both from Cell Signaling) for 1 h in the dark at room temperature followed by DyLight649-conjugated AffiniPure Goat At-Rabbit IgG (Jackson ImmunoResearch) for 15 min at 4°C. PBS with 0.5% BSA (Biomol) was used for incubation and washing steps of intracellular antibody stainings. At least 50,000 cells were acquired using a Canto-II (BD Biosciences) with DIVA software (BD Biosciences) and were further analyzed using the FlowJo 10.4.2 software (Tree Star).

### Measurement of antigen uptake by flow cytometry or multispectral imaging flow cytometry

MoDCs (5 × 10^5^) were seeded in a 48-well plate and stimulated for 24 h with 3 μg/mL Sa lysate or 100 ng/mL LPS with or without PSMα3 (10 μM) or PSMα3 alone prior to the incubation with Ovalbumin (OVA)-AlexaFluor647 (5 μg/mL, Invitrogen) together with PSMα2 FITC (0.5 μM) for 30 min at 37°C, 5% CO_2_. Unspecific binding of OVA/PSMα2 was assessed by incubating the cells on ice. Cells were washed twice with ice-cold PBS containing 2% FBS. Subsequently, cells were blocked and stained with ZombieAqua (BioLegend), HLA-DR BV650 (L243, BioLegend), HLA-DR APC-Cy7 (L243, BioLegend) and CD11c PE-Cy7 (Bu15, BioLegend) as described above and analyzed by flow cytometry or by multispectral imaging flow cytometry. For the latter, images of 10,000 living moDCs were acquired using the Image-Stream mkII (Amnis) with the INSPIRE instrument controller software. The data were analyzed using the IDEAS analysis software (Merck Millipore).

### Lactate dehydrogenase release

MoDCs (2 × 10^5^) were seeded in a 96-well plate and treated with Triton X100 (1%; Sigma-Aldrich), DMSO (2%, Fluka), PSMα2 (10 μM), PSMα3 (2.5, 5, 7.5, and 10 μM), δ-Toxin (10 μM) or OVA (10 μg, Sigma-Aldrich) for 10 min at 37°C, 5% CO_2_. Cell death was determined using 7-aminoactinomycin D (7-AAD, Biomol) staining and acquisition on a Canto II flow cytometer. Supernatants were used for the analysis of lactate dehydrogenase (LDH) release using the Cytotoxicity Detection Kit (Roche) according to the manufacturer's instructions. Absorbance was measured at 492 nm and 620 nm over a period of 1 h with an interval of 5 min using the Spark 10 M microplate reader (Tecan).

### T-cell assay

MoDCs (5 × 10^4^) were seeded in a 96-well plate and stimulated with 3 μg/mL Sa lysate or 100 ng/mL LPS with or without PSMα3 (10 μM) or PSMα3 alone for 24 h. For some experiments moDCs were pre-treated with 200 μM 1-Methyl-D-tryptophan (1-DMT) for 1 h prior to the stimulation. Human naïve CD4^+^ T cells were isolated from PBMCs using the MojoSort™ Human CD4 Naïve T Cell Isolation Kit (BioLegend) according to the manufacturer's instructions. For the magnetic cell separation, a LS column (Miltenyi Biotech) was placed into the QuadroMACS Separator (Miltenyi Biotech) and rinsed with MACS buffer (PBS containing 0,5% BSA (Biomol) and 2 mM EDTA). The cell suspension was applied to the column, and the column was washed three times with 3 mL MACS buffer. The untouched naïve CD4^+^ T cells were collected in the flow through. The purity of isolated (naïve) CD4^+^ T cells was always ≈85% (Figure S4). The naïve CD4^+^ T cells were labeled with CFSE (5 μM, BioLegend) according to the manufacturer's instructions. 2 × 10^5^ T cells diluted in 100 μL T cell medium [RPMI1640 (Merck), 10% FBS (Sigma), 2 mM L-Glutamine (Life Technologies), 100 U/mL penicillin-streptomycin (Life Technologies), 1 × non-essential amino acids (Merck), 1 mM sodium pyruvate (Merck), 10 mM HEPES (Biochrom) and 50 μM 2-mercaptoethanol (Roth)] were added to the moDCs. To investigate whether secreted factors from DCs upon PSM-treatment mediate T_reg_ priming, T cells were co-cultured with untreated moDCs adding conditioned medium from LPS or LPS + PSMα3 stimulated DCs. In a second assay moDCs treated as described above were splitted using Accutase (Sigma-Aldrich) for 5 min at room temperature and again sowed with either fresh or conditioned DC medium (TLRL or TLRL + PSMα3). In some conditions, DC were fixed with 1% paraformaldehyde for 10 min at 4°C to address the impact of newly secreted factors on T_reg_ priming by DCs. 3–4 d after co-culture T cells were blocked with IgG from human serum for 15 min at room temperature and subsequently stained with ZombieAqua, CD4 APC-Vio770 (REA623, Miltenyi), CD3 Pacific Blue (SK7, BioLegend), CD25 PE-Cy7 (BC96, eBiosciences), CD127 PE (eBioRDR5, eBiosciences) and CD45RA BV605 (HI100, BioLegend) for 20 min at 4°C. For intracellular staining, cells were fixed and permeabilized with the Foxp3/ Transcription Factor Staining Buffer Set (eBiosciences), blocked and stained with CD4 APC-Vio770, CD3 Pacific Blue, FoxP3 AlexaFluor647 (259D, BioLegend), T-bet PE-Dazzle 594 (4B10, BioLegend), GATA3 PerCP-Cy5.5 (16E10A23, BioLegend) and RORγt BV650 (Q21-559, BD Biosciences) for 45 min at 4°C. FACS buffer was used for all incubations and washing steps for the extracellular staining, and 1 × permeabilization buffer (Foxp3/Transcription Factor Staining Buffer Set (eBiosciences) was used for all incubations and washing steps for the intracellular staining. At least 70,000 cells were acquired using an LSR Fortessa flow cytometer (BD Biosciences) with the DIVA software (BD Biosciences) and were further analyzed using FlowJo 10.4.2 software (Tree Star).

### Autologous T-cell assay

CD14^+^ cells from PBMCs of patients with TH17-associated autoimmune diseases were isolated by MACS using CD14 MicroBeads (Miltenyi Biotech) and plated in a tissue-treated 6-well plate (1.3 × 10^6^ cells per well) in DC medium containing 50 ng IL-4 and 100 ng GM-CSF for 6 d to generate moDCs. The remaining CD14^−^ cells were frozen at −80°C in RPMI1640 supplemented with 20 % FBS and 10% DMSO. After 6 d moDCs (5 × 10^4^) were seeded in a 96-well plate and stimulated with 100 ng/mL LPS with or without PSMα3 (10 μM) for 24 h. The CD14^−^ cells were thawed and used to isolate CD4^+^ T cells by MACS with CD4 MicroBeads (Miltenyi Biotech). The CD4^+^ T cells were labeled with CFSE (5 μM, BioLegend) according to the manufacturer's instructions and 2 × 10^5^ T cells diluted in 100 μL T cell medium were added to the moDCs. 3–4 days after co-culture T cells were stained as above and iT_regs_ were analyzed by flow cytometry using an LSR Fortessa flow cytometer (BD Biosciences) with the DIVA software (BD Biosciences) and were further analyzed using FlowJo 10.4.2 software (Tree Star).

### Cytokine production in the moDC T cell co-culture

Fifty microliter cell culture supernatants from the T cell assay were taken on day 1, 2 and 3 and cytokine production from 15 μL was analyzed by performing bead-based immunoassays in a 96-well plate [LEGENDplex human B cell Panel (13-Plex) and LEGENDplex Free Active/Total TGF-β1 (BioLegend)] according to the manufacturer's instructions, using the Lyric flow cytometer with autosampler (BD Bioscience).

### T cell suppression assay

MoDCs (2 × 10^5^) were seeded in a 48-well plate and stimulated with 100ng/mL LPS and 10 μM PSMα3 for 24 h. Human CD4^+^ T cells were isolated from PBMCs using the human CD4 MicroBeads Kit (Miltenyi) according to the manufacturer's instructions using LS columns. 8 × 10^5^ T cells were added to the stimulated moDCs and cultured for 4 d at 37°C, 5% CO_2_. T cells were stained with CD4 APC-Vio770, CD25 PE-Cy7, CD127 PE and CD45RA PerCP (HI100, BioLegend) as described above and dead cells were excluded using DAPI (Sigma-Aldrich,16,7 ng/mL). T_regs_ were purified by FACS sorting using an ARIA IIu cell sorter (BD Bioscience), according to the surface molecule expression (CD4^+^CD127^−^CD25^hi^CD45RA^−^, see Figure S7). CD4^+^ T cells isolated from PBMCs from a different donor were used as T effector (T_eff_) cells purified with CD4 MicroBeads Kit (Miltenyi). The T_effs_ were labeled with CFSE (5 μM), and 8 × 10^4^ cells were seeded in a 96- well plate in T cell medium together with the indicated numbers of sorted T_regs_. For T cell activation of the T_effs_ Dynabeads (Human T-Activator CD3/CD28Proliferation; Gibco) were added according to the manufacturer's instructions. The proliferation of T_effs_ was assessed after 3 d by flow cytometry. Dead cells were excluded by staining the cells with ZombieAqua. 20,000 cells were acquired at the Canto II with the DIVA software (BD Biosciences) and were further analyzed using the proliferation tool in FlowJo 10.4.2 software (Tree Star).

### Statistical analysis

Statistical analysis was performed using GraphPad Prism 7.0a software (GraphPad, San Diego, CA). Statistical differences were determined using one-way ANOVA with Turkey's posttest in case data were normally distributed (Shapiro-Wilk normality test). Otherwise, data were analyzed using the Kruskal-Wallis nonparametric test. The differences were considered as statistically significant if *p* < 0.05 (^*^), *p* < 0.005 (^**^), *p* < 0.001 (^***^), or *p* < 0.0001 (^****^).

## Results

### PSMs modulate surface molecule expression of TLR-treated moDCs

**Figure 1:**
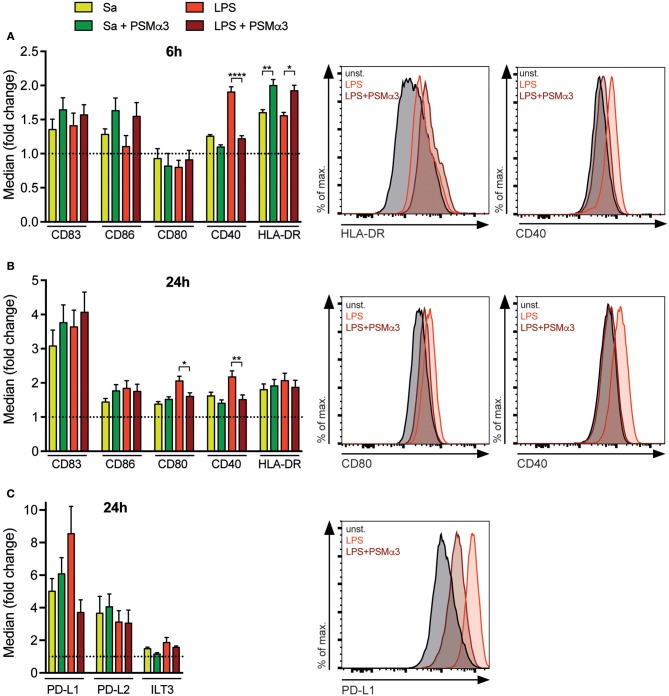
PSMs modulate surface molecule expression of TLR-treated moDCs. MoDCs were treated with Sa lysate or LPS with or without PSMα3 for 6 h **(A)** or 24 h **(B,C)** and analyzed by flow cytometry. MoDCs were characterized as living CD11c^+^HLA-DR^+^ cells and the expression of the indicated costimulatory **(A,B)** and inhibitory molecules **(C)** was determined. The graphs show the mean fluorescence intensity of the respective marker expression as fold change of untreated cells. The graphs show *n* ≥ 3 independent experiments (mean ± SEM) performed in triplicates. Representative histogram overlays of HLA-DR and CD40 after 6 h **(A)**, CD80, CD40 **(B)**, and PD-L1 **(C)** after 24 h. *p < 0.05, **p < 0.005 or ****p < 0.0001, one-way ANOVA with Turkey's posttest or Kruskal-Wallis with Dunn's posttest.

The maturation of DCs is an essential event for successful T-cell activation comprising the upregulation of maturation markers (e.g., CD83 and HLA-DR), co-stimulatory (e.g., CD80, CD86, CD40) and co-inhibitory molecules (e.g., PD-L1, PD-L2 or ILT3). To investigate if the Sa-derived toxin PSMα3 has an impact on DC maturation, moDCs were treated with either the TLR2 ligand Sa lysate (12) or the TLR4 ligand LPS with or without PSMα3. Surface marker expression was analyzed by flow cytometry after 6 or 24 h (Gating see Figure S1). All surface molecules analyzed were increased upon TLRL treatment compared to untreated moDCs at 6 and 24 h with the exception of CD80 after 6 h (Figure 1) confirming DC maturation (mDCs – mature DCs). Simultaneous treatment of moDCs with TLRLs and PSMα3 for 6h revealed a tendency, but no significant increase of the maturation marker CD83 and CD86 compared to treatment with TLRLs alone (Figure 1A). In contrast, HLA-DR was significantly increased 6h (Sa *p* = 0.0081; LPS *p* = 0.0226) after TLRL treatment in combination with PSMα3 whereas CD40 was less expressed on mDCs treated with LPS in combination with PSMα3 compared to LPS treated cells after 6 h (LPS *p* < 0.0001) and 24 h (LPS *p* < 0.0049) (Figures 1A,B). Similarly, CD80 up-regulation on TLR4-stimulated mDCs was impaired when the cells were treated with PSMα3 for 24 h (LPS *p* = 0.0135) (Figure 1B). The analysis of co-inhibitory molecule expression revealed no significant differences after 24 h; however, PSMα3 showed a tendency to prevent the upregulation of the co-inhibitory molecule PD-L1 in TLR4-stimulated moDCs (LPS *p* = 0.0615) (Figure 1C). In summary, PSMα3 enhanced the early upregulation of HLA-DR on DCs but prevented that of the co-stimulatory molecules CD40 and CD80 especially upon TLR4 stimulation.

### PSMs impair pro- and anti-inflammatory cytokine secretion by TLR4-treated moDCs

**Figure 2:**
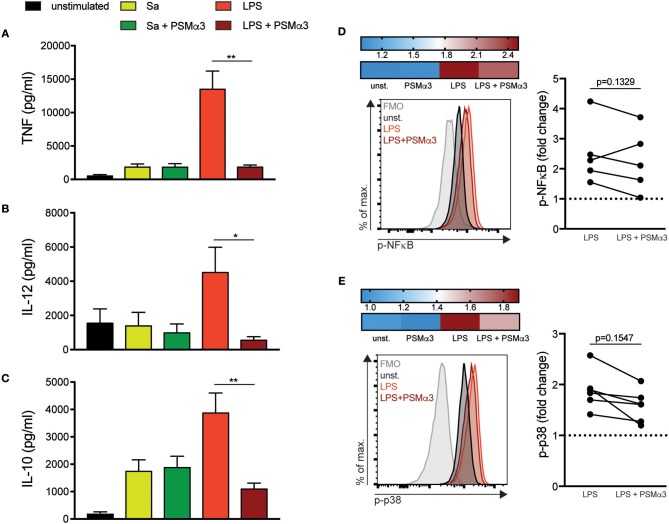
PSMs impair pro- and anti-inflammatory cytokine secretion by moDCs. **(A–C)** MoDCs were treated with Sa lysate or LPS with or without PSMα3 for 6 h **(A)** or 24 h **(B,C)**. The cell culture supernatants were collected and analyzed for TNF **(A)**, IL-12 **(B)** and IL-10 **(C)** production by sandwich ELISA. The graphs show *n* ≥ 10 independent experiments (mean ± SD/SEM) performed in triplicates. **(D,E)** MoDCs were treated with LPS with or without PSMα3 or PSMα3 alone for 60 min. Thereafter, the cells were stained extracellularly with anti-CD11c and anti-HLA-DR antibodies, followed by fixation, permeabilization and subsequent intracellular staining using anti-phospho-NF-κB **(D)** and anti-phospho-p38 antibodies **(E)**. Representative histogram overlays of p-NF-κB **(D)** and p-p38 **(E)** in DCs (gated on CD11c^+^HLA-DR^+^ cells). The heat map shows fold-change of p-NF-κB **(D)** and p-p38 **(E)** normalized to untreated DCs (unst.). The graphs show the statistical analysis of p-NF-κB **(D)** and p-p38 **(E)** from *n* = 5 different donors. *p < 0.05 or **p < 0.005, Kruskal-Wallis with Dunn's posttest or one-way ANOVA with Turkey's posttest.

Stimulation of TLRs not only leads to DC maturation but also to the expression of cytokines to initiate an immune response. Previously, we showed that PSMα3 impaired the pro-inflammatory cytokine production triggered by various TLRLs in mouse BM-DCs, whereas it induced the expression of the anti-inflammatory cytokine IL-10 (12, 18). Therefore, cell culture supernatants from moDCs treated with Sa lysate or LPS with or without PSMα3 were analyzed after 6 h for TNF (Figure 2A) and after 24 h for IL-12 (Figure 2B), IL-10 (Figure 2C) or TNF production (Figure S2A). TLRL treatment led to an overall induction of cytokine secretion with the exception of IL-12 (Figure 2B) in TLR2-ligand treated mDCs (Figure 2). The production of TNF, IL-12 and IL-10 was impaired by PSMα3 in LPS treated mDCs (TNF 1.800 vs. 13.500 pg/ml, *p* = 0.0082; IL-12 550 vs. 4.500 pg/ml, *p* = 0.021; IL-10 1090 vs. 3850 pg/ml, *p* = 0.0080) (Figure 2) the latter in a concentration dependent manner (Figure S2B), but not in Sa lysate treated mDCs. The treatment of DCs with PSMα3 in combination with other TLR-ligands like Pam2CSK4, Pam3CSK4, LTA, Flagellin, CpG, and Imiquimod showed no significant differences in the production of TNF and IL-10 compared to TLR-L treatment alone (Figure 2 and Figure S2C). In summary, PSMα3 inhibited pro- as well as anti-inflammatory cytokine production by LPS-treated mDCs.

### PSMs impair NF-κB and p38 phosphorylation in LPS-treated moDCs

To investigate the signaling pathways involved in the cytokine modulation by PSMs, DCs were treated with LPS in the presence or absence of PSMα3 for 60 min and phosphorylation of NF-κB p65 (p-NF-κB) (Figure 2D) and p38 MAPK (p-p38) (Figure 2E) was analyzed by flow cytometry. Treatment of DCs with PSMα3 did not affect p- NF-κB or p-p38 compared to untreated DCs (Figures 2D,E heatmap), whereas after LPS treatment a 2.5-fold and 2-fold increase of p- NF-κB and p-p38, respectively, was observed compared to untreated DCs (Figures 2D,E). DCs incubated with LPS combined with PSMα3 revealed a 2.0-fold and 1.5-fold increase of NF-κB or p38 phosphorylation (Figures 2D,E). Analyzing signaling pathways in moDCs from 5 donors revealed by trend a reduced, but not significant, NF-κB or p38 phosphorylation upon PSMα3 treatment. These data indicate that impaired phosphorylation of both NF-κB and p38 pathways by PSMα3 may account for impaired cytokine production as well as co-stimulatory molecule expression by moDCs upon LPS treatment.

### PSMs inhibit antigen uptake by moDCs

**Figure 3:**
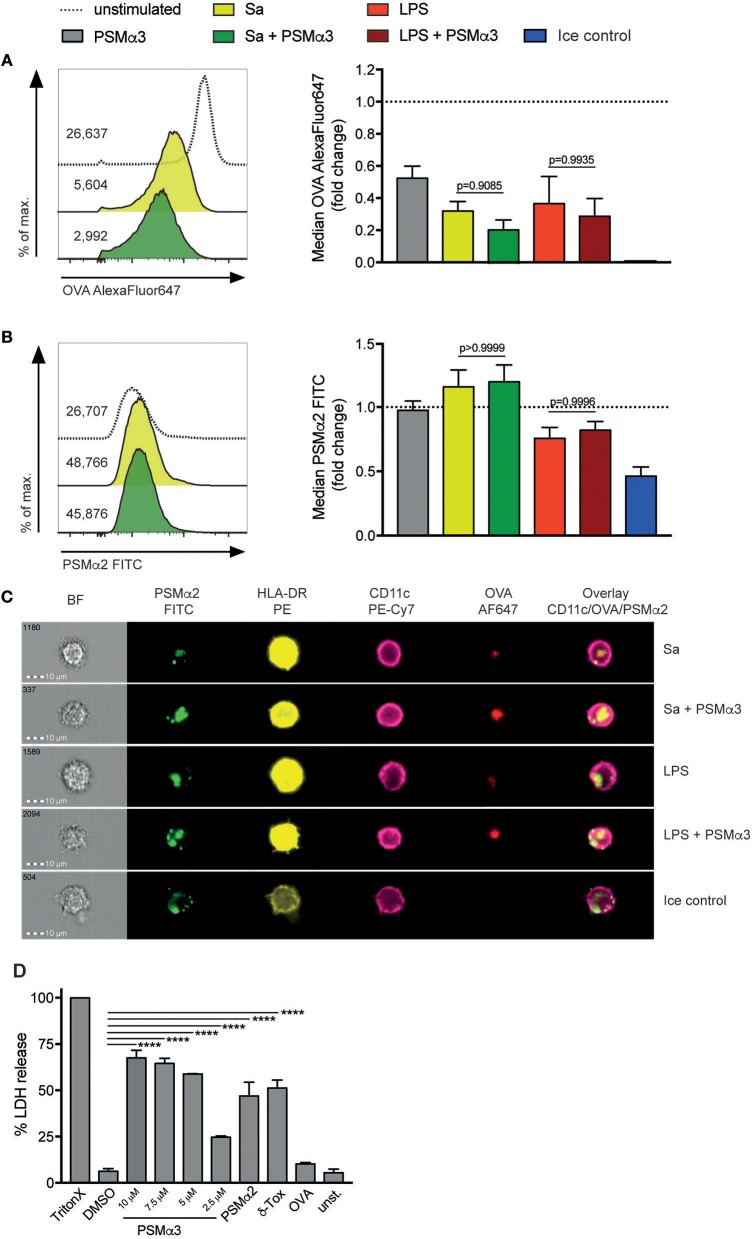
PSMs inhibit antigen uptake by moDCs and penetrate the membrane via transient pore formation. **(A–C)** MoDCs treated for 24 h with Sa lysate or LPS with or without PSMα3 or PSMα3 alone were incubated with AlexaFluor647-labeled OVA and FITC-labeled PSMα2 at 37°C or on ice for 30 min. **(A,B)** The uptake of OVA and PSMα2 by living CD11c^+^HLA-DR^+^ moDCs was assessed by flow cytometry. The histogram overlays (left) and the bar graphs (right, collected data) show the mean fluorescence intensity of OVA-AlexaFluor647 **(A)** or PSMα2-FITC **(B)**, in case of the collected data as fold change of unstimulated cells (*n* ≥ 5, performed in triplicates, mean ± SEM). **(C)** The localization of OVA-AlexaFluor647 and PSMα2-FITC in CD11c^+^HLA-DR^+^ moDCs was analyzed by multispectral imaging flow cytometry (one representative experiment of *n* = 3 independent experiments; mean ± SEM). Representative bright field (BF) and fluorescence images of moDCs are shown for the different treatments. **(D)** MoDCs were either not treated (unst.) or incubated with 1% Triton X-100, 2% DMSO, PSMα3 (2.5 μM, 5 μM, 7.5 μM or 10 μM), 10 μM PSMα2, 10 μM δ-Toxin or 5 μg/mL OVA for 10 min. The LDH release was determined in the cell culture supernatants (one representative experiment of *n* = 3 independent experiments; mean ± SEM). ****p < 0.0001, one-way ANOVA with Turkey's posttest or Kruskal-Wallis with Dunn's posttest.

Antigen uptake is a pivotal task of DCs and necessary for T-cell activation. To elucidate possible effects of PSMs on this event, moDCs were treated with or without either Sa lysate or LPS in the presence or absence of PSMα3 for 24 h. Subsequently cells were incubated with the fluorescently-labeled (AlexaFluor647) model antigen OVA in combination with FITC-labeled PSMα2 for 30 min. Immature DCs (iDCs – immature DCs) take up OVA mainly by clathrin-mediated endocytosis and macropinocytosis, whereas mature DCs retain antigen uptake by receptor-mediated endocytosis (12, 19–21). This effect was observed when moDCs matured after treatment with either Sa lysate or LPS by ~68 or ~63%, respectively (Figure 3A). OVA uptake was reduced by ~48% after solely treatment with PSMα3 (*p* < 0.0001) (Figure 3A), showing that iDCs are affected in their task to take up antigen by PSMs. A trend to further impaired antigen uptake by TLRL and PSMα3 treated moDCs was observed (Sa *p* = 0.6603, LSP *p* = 0.9054) (Figure 3A). Incubation of iDCs with OVA on ice was used to exclude unspecific binding (Figure 3A) thereby preventing remodeling of the actin cytoskeleton, which is required for clathrin-mediated endocytosis and micropinocytosis (22). In conclusion, PSMα3 affects moDCs in their antigen uptake capacity.

### PSMs penetrate the membrane of moDCs via transient pore formation

Previously, we and others showed that PSMs form transient pores into the cell membrane thereby entering the cytosol (10, 15). To address whether PSMs are internalized by human moDCs via mechanisms of antigen uptake or by pore formation iDCs were incubated with fluorescently-labeled PSMα2 as described above and analyzed by flow cytometry and multispectral imaging flow cytometry. Despite the uptake of OVA-AlexaFluor647, PSMα2-FITC was observed in moDCs regardless of their maturation status (Figure 3B). Moreover, PSMα2-FITC was also detected in moDCs incubated on ice but reduced by ~54% (*p* = 0.0003), indicating that the uptake was not an active process requiring actin remodeling (Figure 3B). These observations were confirmed by multispectral imaging flow cytometry, showing PSMα2-FITC located at the membrane when actin-cytoskeleton rearrangement is blocked (ice control) as well as co-localized with OVA most likely in the phagosome when moDCs were incubated at 37°C regardless of the treatment (Figure 3C).

Transient pore formation in the membrane of moDCs mediated by PSMα3 was demonstrated by measuring L-lactate dehydrogenase (LDH) in the supernatant of moDCs treated with PSMα2 various concentrations of PSMα3 (Figure 3D). Triton-X, which disrupts the membrane completely as well as δ-Toxin, which is known to form transient pores (23) were used as positive controls (Figure 3D). Neither treatment of moDCs with OVA, the peptide-dissolvent DMSO or medium alone had any effects on LDH release (Figure 3D), whereas PSMα3 significantly induced LDH release in a concentration-dependent manner. Further, flow cytometric analysis of the moDCs showed that apart from treatment with Triton-X, none of the reagents affected their viability (Figure S3). Together the data show that PSMs enter moDCs via transient pore formation without cytolytic effects.

### Polarization of T helper1 cells is impaired by PSMs

**Figure 4:**
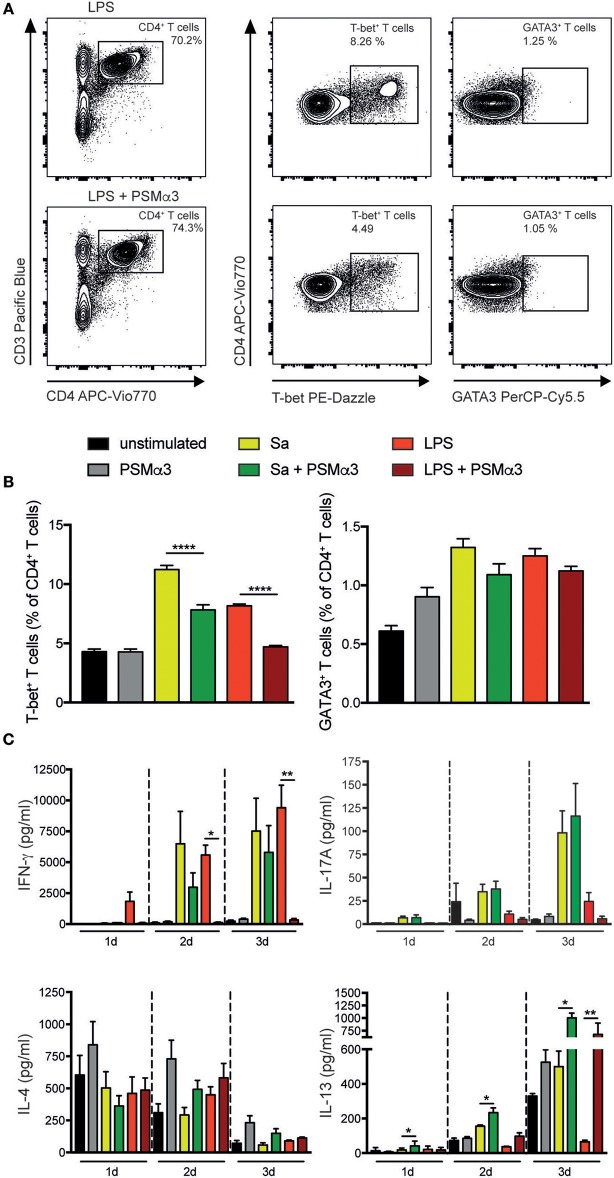
Polarization of Th1 is impaired by PSMs. MoDCs treated for 24 h with Sa lysate or LPS with or without PSMα3 or PSMα3 alone were co-cultured with CFSE-labeled naïve CD4^+^ T cells for 3 d. The different Th subsets were analyzed by flow cytometry; Th1 cells were characterized as CD3^+^CD4^+^T-bet^+^ T cells, Th2 cells were characterized as CD3^+^CD4^+^GATA3^+^ T cells **(A)**. **(B)** The bar graphs show the frequency of T-bet^+^ Th1 and GATA3^+^ Th2 cells from CD4^+^ T cells (one representative of *n* = 4 independent experiments performed in triplicates, mean ± SEM). **(C)** Cell culture supernatants from the DC T cell co-culture were collected after 1 d, 2 d or 3 d and analyzed for Th-associated cytokine production (*n* ≥ 3 performed in triplicates; mean ± SEM). *p < 0.05, **p < 0.005, or ****p < 0.0001, one-way ANOVA with Turkey's posttest or Kruskal-Wallis with Dunn's posttest.

DCs play a crucial role in the activation and polarization of T cells. Not only the direct interaction, but also the local cytokine milieu is important for the outcome and distinct transcription factors control the differentiation of the T cell subsets (24, 25). To assess the impact of PSMα3-treated moDCs on the polarization of T helper (Th) cell subsets, moDCs were treated with or without Sa lysate or LPS in combination with PSMα3 for 24 h. Subsequently, the cells were co-cultured with naive CD4^+^ T cells for 3–4 d. Flow cytometry analysis of the Th subsets showed that TLRL-treated mDCs primed significantly more T-bet^+^ Th1 cells whereas mDCs co-treated with PSMα3 prevented Th1 priming (Sa 11.2 vs. 7.8%, *p* < 0.0001; LPS 8.2 vs. 4.7%, *p* < 0.0001) (Figures 4A,B). In contrast, stimulation of iDCs with PSMα3 alone did not show any difference compared to untreated cells (Figure 4B), indicating that PSMα3 without TLRL has no influence on the priming capacity of moDCs. Analyzing Th2 cells by GATA3 expression after co-culture with moDCs treated with TLRL alone or in combination with PSMα3 revealed the same tendency as T-bet expression, but no significant differences (Sa 1.32 vs. 1.1%, *p* = 0.27; LPS 1.25 vs. 1.1%, *p* = 0.8) (Figures 4A,B and Figure S6B). However, no difference in the frequency of RORγt^+^CD4^+^ T cells was observed (data not shown).

Cytokine secretion analysis of the major Th1, Th2, and Th17 cytokines in co-culture after 1–3 days showed that IFN-γ secretion was completely prevented when TLR4 ligand-treated mDCs were co-treated with PSMα3 (LPS day 1 1.800 vs. 80 pg/ml, *p* = 0.5617; day 2 5.600 vs. 120 pg/ml, *p* = 0.0146, day 3 9.400 vs. 330 pg/ml, *p* < 0.0001), which in part, but not significant, was also seen for TLR2-stimulated mDCs (Sa day 1 60 vs. 40 pg/ml, p>0.999; day 2 6.500 vs. 3.000 pg/ml, *p* = 0.2534, day 3 7.500 vs. 5,800 pg/ml, *p* = 0.9105) (Figure 4C). Comparable results, but without significance, were observed for IL-17A secretion at day 2 and 3 by TLR4 ligand-treated mDCs together with PSMα3 (LPS day 2 10.8 vs. 5.1 pg/ml, *p* = 0.9658, day3 24.4 vs. 5.7 pg/ml, *p* = 0.4336). Similarly, no significant effects of PSMα3 were observed for IL-17A secretion after co-culture of CD4^+^ T cells with mDCs treated with Sa lysate. Further, IL-4 secretion showed no difference after treatment with either Sa lysate or LPS, respectively (Figure 4C). However, the expression of the closely related cytokine IL-13 significantly increased 3 days after coculture of CD4^+^ T cells with moDCs treated with TLRLs and PSMα3 (Sa 500 vs. 1.000 pg/ml, *p* = 0.0327; LPS 65 vs. 680 pg/ml, *p* = 0.0057) (Figure 4C). This Th cytokine expression is connected with the impaired production of pro-inflammatory cytokines in co-cultures of CD4^+^ T cells with moDCs treated with TLRLs and PSMα3 (Figures S5A–C).

Thus, PSMα3-treated TLR2- and TLR4- stimulated mDCs decreased the frequency of T-bet^+^ Th1 cells, as well as IFN-γ secretion.

### PSMα3-treated moDCs induce T_reg_ priming via direct cell interaction and IDO production

**Figure 5:**
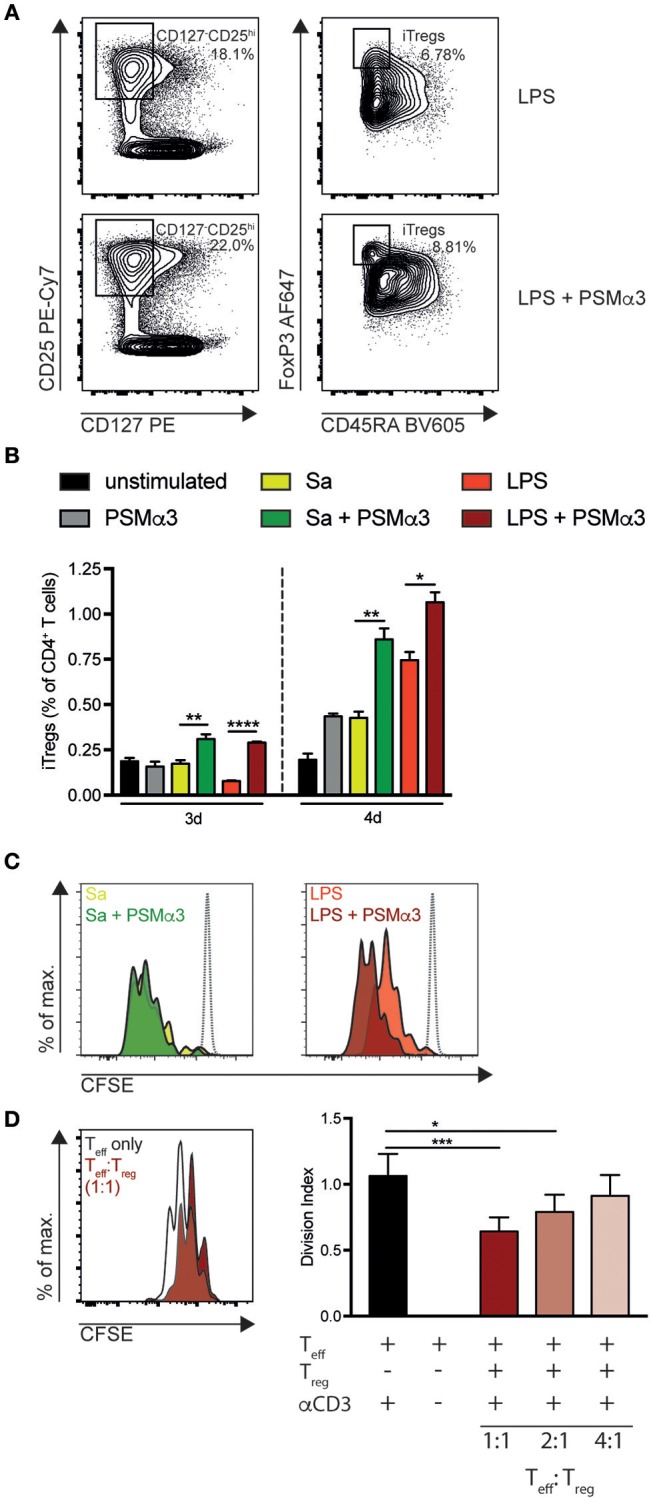
PSMα3-treated moDCs increase the proliferation and frequency of iT_regs_. **(A–C)** MoDCs treated for 24 h with Sa lysate or LPS with or without PSMα3 or PSMα3 alone were co-cultured with CFSE-labeled naïve CD4^+^ T cells for 3 d or 4 d. iT_regs_ were analyzed by flow cytometry and characterized as CD4^+^CD127^−^CD25^hi^CD45RA^−^Foxp3^hi^ cells **(A)**. The frequency of iT_regs_ from CD4^+^ T cells **(B)** and the proliferation of iT_regs_ **(C)** co-cultured with TLRL-only or in combination with PSMα3-treated mDCs was analyzed (n ≥ 5 performed in triplicates, mean ± SEM). Proliferation was compared to T cells cultured without DCs (gray dotted line). **(D)** Allogenic suppression assay of CFSE-labeled T_effs_ cultured in different ratios with or without sorted T_regs_ (CD4^+^CD127^−^CD25^hi^CD45RA^−^, Figure S7) from DC T cell co-culture described in **(A)**. Proliferation of the CFSE-labeled T_effs_ with or without activation via αCD3-beads was assessed after 3 d (*n* = 2 performed in triplicates; mean ± SEM). *p < 0.05, **p < 0.005, ***p < 0.001, or ****p < 0.0001, one-way ANOVA with Turkey's posttest or Kruskal-Wallis with Dunn's posttest.

Previously, our group showed that PSMα3 primes mouse tDCs thereby enhancing the frequency and proliferation of T_regs_ (10, 12). Further, as PSMα3 treatment of moDCs attenuated the priming of Th1 cells, we investigated whether PSMα3-treated moDCs also increased T_reg_ priming. Therefore, moDCs were treated with or without Sa lysate or LPS in combination with PSMα3 for 24 h. Next, the cells were co-cultured with naïve CFSE-labeled CD4^+^ T cells for 3–4 d and analyzed by flow cytometry. Newly primed T_regs_ were characterized as CD4^+^CD127^−^CD25^hi^CD45RA^−^FoxP3^hi^ induced T_regs_ [iT_regs_; (26)]. PSMα3 increased the frequency of iT_regs_ upon co-culture with moDCs after TLR2 or TLR4 ligand treatment (Sa day 3 0.17 vs. 0.31%, *p* < 0.0033; Sa day 4 0.43 vs. 0.86%, *p* = 0.0054; LPS day 3 0.08 vs. 0.29%, *p* < 0.0001; LPS day 4 0.75 vs. 1.07%, *p* = 0.0204) (Figures 5A,B). Treating CD4^+^ T cells with PSMα3 in combination with TLRLs without moDCs did not result in CD4^+^ T cell activation or priming of iT_regs_ (Figure S6).

Further, these iT_regs_ showed a greater proliferation potential than the iT_regs_ primed without PSMα3 (Figure 5C). To test the functionality of these iT_regs_, we sorted iT_regs_ (CD4^+^CD127^−^CD25^hi^CD45RA^−^) after 4 d of co-culture with LPS + PSMα3-treated moDCs (Figure S7). Sorted iT_regs_ were again cultured with CFSE-labeled CD4^+^ T cells (T_eff_) in the presence of anti-CD3/CD28 coated beads to induce T_eff_ proliferation for 4 d. T_regs_ significantly decreased T_eff_ proliferation analyzed by CFSE-dilution in a concentration-dependent manner (1:1 reduction ~38%, *p* = 0.0004; 1:2 reduction ~25%, *p* = 0.0176; 1:4 reduction ~12%, *p* = 0.2603) (Figure 5D), demonstrating that PSMα3-induced iT_regs_ suppress T_eff_ proliferation.

**Figure 6:**
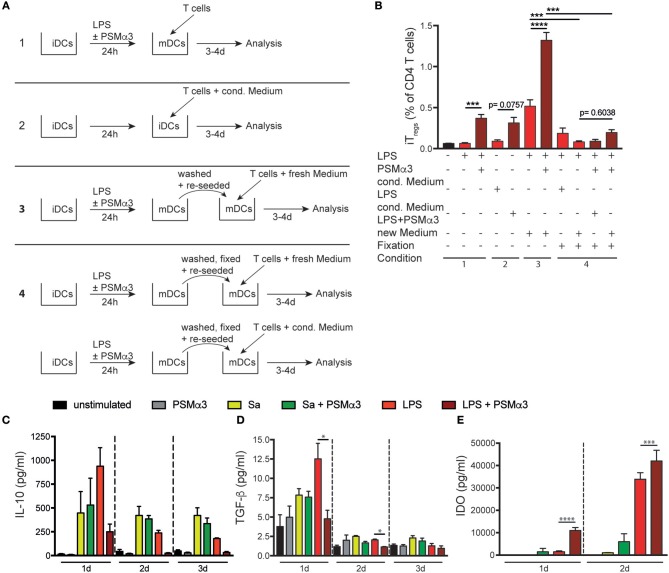
PSMα3-treated moDCs increase the proliferation and frequency of iT_regs_ by direct interaction and IDO production. **(A)** Schematic overview of the experimental setting: T cells were co-cultured for 3–4 d (1) with mDCs (LPS or LPS + PSMα3 for 24 h), with (2) untreated moDCs (iDCs) in the presence of conditioned medium from LPS or LPS + PSMα3 treated DCs, with (3) washed and re-seeded mDCs together with new medium or (4) with fixed moDCs together with either new medium or conditioned medium. **(B)** The bar graphs show the frequency of iT_regs_ from CD4^+^ T cells analyzed by flow cytometry from the experiments described in **(A)** (one representative of *n* ≥ 3 independent experiments performed in triplicates). **(C,D)** Cell culture supernatants from the DC T cell co-culture (*n* ≥ 3 performed in triplicates; mean ± SEM) were analyzed for IL-10 **(C)** and TGF-β production **(D)** after 1 d, 2 d or 3 d by a bead-based immunoassay. **(E)** Cell culture supernatants (*n* = 2 performed in triplicates; mean ± SEM) from moDCs treated with Sa lysate or LPS with or without PSMα3 were analyzed for IDO production by sandwich ELISA. *p < 0.05, ***p < 0.001 or ****p < 0.0001, one-way ANOVA with Turkey's posttest or Kruskal-Wallis with Dunn's posttest.

To investigate whether secreted factors from DCs upon PSM-treatment mediate iT_reg_ priming, T cells were co-cultured with iDCs simultaneously adding medium from LPS or LPS with PSMα3-treated moDCs (conditioned medium) (Figure 6A condition 2). As described above, PSMα3 increased the frequency of iT_regs_ upon co-culture of naïve CD4^+^ T cells with moDCs (Figure 6A condition 1 and Figure 6B). A similar increase of iT_regs_ was mediated by iDCs incubated with conditioned medium from LPS + PSMα3, whereas LPS conditioned medium had no effect (0.31% compared to 0.09%, respectively, *p* = 0.0757) Figure 6A condition 2 and Figure 6B). These observations show that PSM-induced secretion of soluble factors by mDCs modulate iDCs to prime iT_regs_.

In another assay DCs were washed 24 h after treatment with LPS or LPS + PSMα3 (mDCs) and further cultured for 3–4 days with CD4^+^ T cells in fresh medium. These conditions revealed an even higher frequency of iT_regs_ (0.52 or 1.32%, respectively; *p* < 0.0001) compared to mDCs without medium change (Figure 6A condition 3 and Figure 6B) and suggest that the interaction of mDCs with CD4^+^ T cells as well as secreted factors produced by mDCs after interaction with T cells are important for iT_reg_ priming.

To further address the impact of direct DC-T-cell interaction on T_reg_ priming, mDCs were fixed and either cultured with naïve CD4^+^ T cells in fresh or conditioned medium. Fixation of mDCs treated with or without PSMα3 led to low frequencies of iT_regs_ after co-culture with naïve CD4^+^ T cells in fresh medium (0.08% vs. 0.2%, *p* = 0.6038) (Figure 6A condition 4 and Figure 6B). Similar results were obtained by the addition of conditioned medium to this co-culture showing that mainly direct interaction of mDCs with naïve CD4^+^ T cells is essential for iT_reg_ induction. However, in this experimental setting we cannot exclude that soluble factors produced by mDCs after DC-T cell interaction are involved in iT_reg_ priming. To address this issue, culture supernatants of mDCs with naïve CD4^+^T cells were analyzed over time for soluble factors, which were shown to be responsible for iT_reg_ priming and proliferation, like TGF-β, IL-10, IL-2, CD40L and IDO. Neither, IL-10 (Figure 6C), TGF-ß (Figure 6D), CD40L nor IL-2 (Figure S5 were increased in the co-culture of PSMα3-treated mDCs with naïve CD4^+^ T cells compared to mDCs independently of the TLRL used. Moreover, T helper cell priming cytokines like IL-4, IL-6, IL-12p70, IL17A and IFN-γ were hardly detectable in the co-culture supernatants of LPS + PSMα3 treated mDCs with CD4^+^ T cells (Figure 4C and Figure S5) as described above.

In contrast, secretion of the enzyme IDO, which is important for T_reg_ differentiation from naïve T cells (8), was significantly increased by mDCs in response to PSMα3 treatment after 1 (11.000 vs. 1.440 pg/ml, *p* < 0.0001) and 2 days (42.000 vs. 33.800 pg/ml, *p* = 0.0006) compared to mDCs alone (Figure 6E). As previously described, LPS treatment alone also led to IDO secretion by moDCs (27). We addressed the impact of IDO on iT_reg_ priming by mDCs using the specific IDO inhibitor 1-Methyl-D-Tryptophan (1-DMT) prior to treatment of iDCs with LPS or LPS + PSMα3. These mDCs were then co-cultured with CD4^+^ T cells and the frequency of iT_regs_ determined as described above. Surprisingly, IDO inhibition revealed increased frequencies of iT_regs_ by 2 fold independently whether cells were treated with LPS + PSMα3 or the inhibitor alone (see Figure S8). No difference in the proliferation of CD4^+^ T cells was observed upon IDO inhibition. Moreover, IDO inhibition had hardly any effect on the frequency of Th1, Th2 and Th17 cells in the culture (data not shown). Thus, the increased IDO production by PSM-treated DCs seems not to be essential for iT_reg_ induction.

Together, these data indicate that PSMα3 modulates moDCs to prime predominantly T_regs_ via mechanisms involving mainly direct DC-T cell interaction in combination with DC-secreted yet unknown factors.

### PSMα3-treated moDCs induce T_regs_ from CD4^+^ T cells of patients with autoimmune diseases

**Figure 7:**
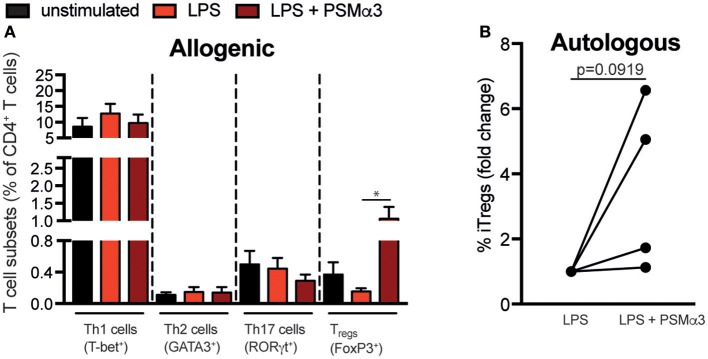
PSMα3-treated moDCs induce T_regs_ from CD4^+^ T cells of patients with autoimmune diseases. **(A)** Allogenic T-cell assay: MoDCs from healthy donors were treated for 24 h with LPS with or without PSMα3 and co-cultured with CFSE-labeled CD4^+^ T cells from patients with Th17-associated spondyloarthritis for 3 d or 4 d. The different T cell subsets were analyzed by flow cytometry. The graph shows the frequency of the T-bet^+^ Th1, GATA3^+^ Th2, RORγt^+^ Th17 cells and CD127^−^CD25^hi^CD45RA^−^Foxp3^hi^ iT_regs_ from CD4^+^ T cells (*n* = 6 patients; mean ± SEM). *p < 0.05, one-way ANOVA with Turkey's posttest or Kruskal-Wallis with Dunn's posttest. **(B)** Autologous T-cell assay: MoDCs from spondyloarthritis patients were treated as described in **(A)** and co-cultured with CFSE-labeled CD4^+^ T cells from the same patient. CD127^−^CD25^hi^CD45RA^−^Foxp3^hi^ iT_regs_ were analyzed by flow cytometry. The graph shows the frequency of iT_regs_ as fold change from LPS-treated cells of 4 different spondyloarthritis patients. Data were analyzed by unpaired student's *T*-test.

In order to address whether PSMα3 can be used for therapeutic approaches by modulating moDCs for iT_reg_ priming in a setting of T cell associated autoimmune diseases, moDCs from healthy donors were co-cultured after treatment with LPS or LPS + PSMα3 with CD4^+^ T cells from patients with spondyloarthritis (Figure 7A) and rheumatoid arthritis (RA) (data not shown). These patients suffer from spondylitis/enthesitis of the spine or peripheral arthritis with pain, morning stiffness and consecutive ankylosing of the spine and/or joint destruction. These diseases display a high frequency of pro-inflammatory Th1 and Th17 cells. As shown for T cells from healthy donors, PSMα3 increased the frequency of iT_regs_ upon co-culture with LPS-treated mDCs by > 10-fold compared to LPS treatment alone (*p* = 0.024), whereas no effects were observed for Th1, Th2, and Th17 priming of T cells from patients with spondylitis (Figure 7A). Moreover, similar, but not significant results (*p* = 0.0919) could be observed in an autologous setting using mDCs and CD4^+^ T cells from patients with spondyloarthritis (Figure 7B and Figure S9). This shows that PSMα3 indeed modulates moDCs to prime iT_regs_ even in allogenic and autologous disease settings, indicating its potential for DC therapy in chronic inflammatory diseases.

## Discussion

There is a need for particular therapeutic approaches preventing or inhibiting immune activation in autoimmune diseases, allograft rejection, allergies, asthma and various forms of hyper-sensitivity. Current therapies, which mainly use nonspecific systemic immunosuppressants, are associated with severe side effects. Thus, *ex vivo* generated tDCs are an attractive alternative to enhance, maintain or restore immunological tolerance.

Here, we addressed the possibility of PSMα3, which is secreted by highly virulent CA-MRSA strains, to induce human tDCs as potential therapeutic for DC vaccination strategies. PSMs form transient pores into the membrane of neutrophils (15), and DCs thereby getting access to the cytosol, in the case of DCs without cell lysis (Figure 3) (10). Molecularly, PSMα3 enhanced the activation of the p38-CREB pathway upon TLR ligation, which in consequence diminished pro-inflammatory cytokine production but induced IL-10 secretion by mouse bone marrow-derived DCs (10). Here, we show that PSMα2 enters human moDCs via endocytosis and transient pore formation and is located in the cytosol as well as close to the membrane. Indeed, NF-κB as well as p38 activation are necessary for DC maturation including upregulation of CD80, CD86, and CD40, but also cytokine production (28–30). We believe that PSMα3 prevents TLR-activation either extracellularly and/or intracellularly thereby inducing tDCs. Direct extracellular interaction of PSMα1–3 with TLR4 was shown to prevent binding of HMGB1 to TLR4 and thus downstream activation of NF-κB (31). Similarly, we here show that PSMα3 prevents activation of NF-κB as well as p38 MAPK signaling. Whether PSMα3 also blocks binding of LPS to TLR4 remains to be shown. Moreover, our findings are in agreement with several studies showing that NF-κB inhibition favors an immature or tolerogenic DC phenotype, which stimulates the expansion of Foxp3 expressing regulatory T cells (32–35).

A hallmark of tDCs is their immature phenotype characterized by low surface levels of MHC class II and costimulatory molecules, such as CD86, CD40, CD54, and PD-L2, but increased expression of TLRs, chemokine receptors and PD-L1. *In vivo* studies showed that the tissue or even tumor microenvironment is important for regulating the development and function of tDCs (36). Although PSMα3 had little influence on TLR–induced up-regulation of HLA-DR, CD83, CD86, and PD-L2, up-regulation of costimulatory molecules CD80, PD-L1, and CD40 was inhibited, preventing full DC-maturation. Likewise, cholera toxin in combination with LPS induced CD80 and CD86 but reduced CD40 and CD54 expression by DCs (37). Mechanistically, the inhibition of TNFα production, which among others regulates CD40 expression, upon synergistic treatment with TLRLs and PSMα3 could explain the impaired up-regulation of CD40 (38). This fits to the fact that the lack of CD40 expression on DCs was shown to be important for the generation of T_regs_ while suppressing primary immune responses (39). Moreover, some studies have shown that phenotypically mature DCs are also able to promote T_regs_ and act superior in activating their suppressor function (40, 41).

In addition, PSMα3 dramatically changed the cytokine secretion pattern of moDCs upon TLRL treatment by preventing the secretion of the pro-inflammatory cytokines TNF, IL-12, but also of anti-inflammatory IL-10, which is produced by DCs in response to TLR-stimulation. This is in contrast to the data observed by mouse bone-marrow derived DC, where IL-10 secretion is even increased upon PSM and TLRL treatment compared to TLRL treatment alone (12). However, PSM-treated moDCs still impair Th1 but promote T_reg_ priming. Tolerogenic DCs are defined by their capacity to induce T_regs_ via production of anti-inflammatory molecules that may be secreted, membrane bound, or both. A variety of studies demonstrated the necessity of IL-10 secretion by tDCs for T_reg_ induction (42–45) and for the maintenance of suppressive T_regs_ upon strong inflammatory signals (12, 44, 46, 47). The data described herein raise the question whether IL-10 secretion is a hallmark of tDCs.

As T-cell differentiation is mainly controlled by cytokines mediating polarizing signals (48) we addressed other factors as mediators for T_reg_ induction. IDO, an immune-regulatory enzyme, which is mainly expressed in DCs, was shown to modulate adaptive immune responses by promoting immune-suppression and tolerance (49–51). IDO expression in DCs is induced either by IFN-γ or by TLR activation via the non-canonical NF- κB pathway. IDO acts through tryptophan (TRP) depletion and production of TRP metabolites thereby inducing differentiation of new T_regs_ from naïve T cells (27, 51–55). PSM treatment of moDCs resulted in an even enhanced IDO expression as compared to Sa lysate or LPS treatment alone. The latter was recently shown to induce IDO together with the transcription factor aryl-hydrocarbon receptor (AhR) (27). However, our results obtained with IDO inhibition by 1-DMT argue against a mechanistically role of IDO in PSM-mediated T_reg_ induction. Thus, it is tempting to speculate that PSMα3 modulated moDCs prime T_regs_ via mechanisms involving predominantly direct DC-T cell interaction in combination with the absence of Th-priming cytokines and probably yet unknown DC-secreted factors.

The data from this study are a proof of concept of the potential use of Sa-derived PSMα3 to induce tolerogenic human DCs with the ability to prime iT_regs_. Especially, when using PSMα3-induced tDCs to prime iT_regs_ in allogenic and autologous settings of CD4^+^ T cells from spondylitis patients. It is believed that tolerogenic DCs may induce tolerance to the pathologic immune responses in a patient without affecting the immune defense against pathogens or tumors. There are two strategies to restore tolerance in autoimmunity: improve the induction and function of tolerogenic DC or generating tolerogenic DC *in vitro* for subsequent administration *in vivo* as cell therapy (13, 56, 57). Different immune-modulatory agents such as dexamethasone, vitamin D3, TNF or IL-10, but also pathogen-derived products have been used in order to modify the phenotype, cytokine profiles and activity of DCs (58–65). Pre-clinical models of arthritis (65), EAE (66), and type 1 diabetes [T1D; (67)] have demonstrated the efficacy of *in vitro* induced tolerogenic DC-based cell therapy.

There is a need for the complete understanding of the mechanisms that control tolerance and immunity in the context of the complexity and heterogeneity of autoimmune diseases, in which multiple cell types are affected and various genetic backgrounds are involved.

## Author contributions

SA, JR, and NA contributed to the conception and design of the study. JR and MG performed the experiments and statistical analysis. SA and JR wrote the first draft of the manuscript. SA, JR, NA, MG, and JH wrote sections of the manuscript. All authors read and approved the submitted version of the manuscript.

### Conflict of interest statement

The authors declare that the research was conducted in the absence of any commercial or financial relationships that could be construed as a potential conflict of interest.

## Abbreviations

APC: antigen presenting cell
BM-DCs: bone-marrow derived DCs
CA-MRSA: community-associated methicillin-resistant Sa
DCs: dendritic cells
IDO: indolamin-2,3-dioxygenase
iT_regs_: induced regulatory T cells
LDH: L-lactate dehydrogenase
moDCs: monocyte-derived DCs
PBMCs: peripheral blood mononuclear cells
PSM: phenol-soluble modulin peptides
Sa: *Staphylococcus aureus*
Sa lysate: *Staphylococcus aureus* cell lysate
tDCs: tolerogenic DCs
Th: T helper cell
TLR: Toll-like receptor
TLRL: Toll-like receptor ligand
T_regs_: regulatory T cells.
