# Supplementary material for: Staphylococcus aureus PSM Peptides Modulate Human Monocyte-Derived Dendritic Cells to Prime Regulatory T Cells
Source: Front Immunol. 2018 Nov 13;9:2603. doi: 10.3389/fimmu.2018.02603 (PMC6282063; doi:10.3389/fimmu.2018.02603)
Supplement: Supplementary file 1 [file Data_Sheet_1.PDF]

Supplementary Material

Staphylococcus aureus PSM peptides modulate human monocyte-derived dendritic cells to prime regulatory T cells

Jennifer R. Richardson[1], Nicole S. Armbruster[1], Manina Günter[1], Jörg Henes[1], Stella E. Autenrieth[1*]

[1]Department of Internal Medicine II, University of Tübingen, Tübingen, Germany

*Correspondence

PD Dr. Stella E. Autenrieth

Stella.Autenrieth@med.uni-tuebingen.de

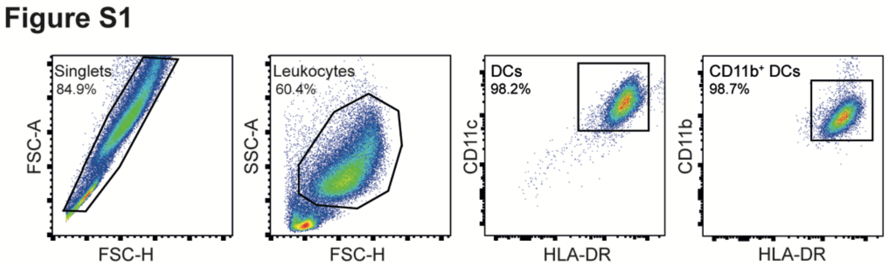

Figure S1: MoDC purity

Isolated PBMCs were plated in DC medium and incubated for 1 h. After the incubation the non-adherent cells were removed by washing and cells were incubated in DC medium containing 50 ng IL-4 and 100 ng GM-CSF for 6 d. Cells were stained with CD11c APC, CD11b BV510 and HLA-DR BV650 antibodies and the purity of moDCs (CD11c$^+$HLA-DR$^+$) and CD11b$^+$ moDCs (parent gate: CD11c$^+$HLA-DR$^+$) was analyzed by flow cytometry (n>5).

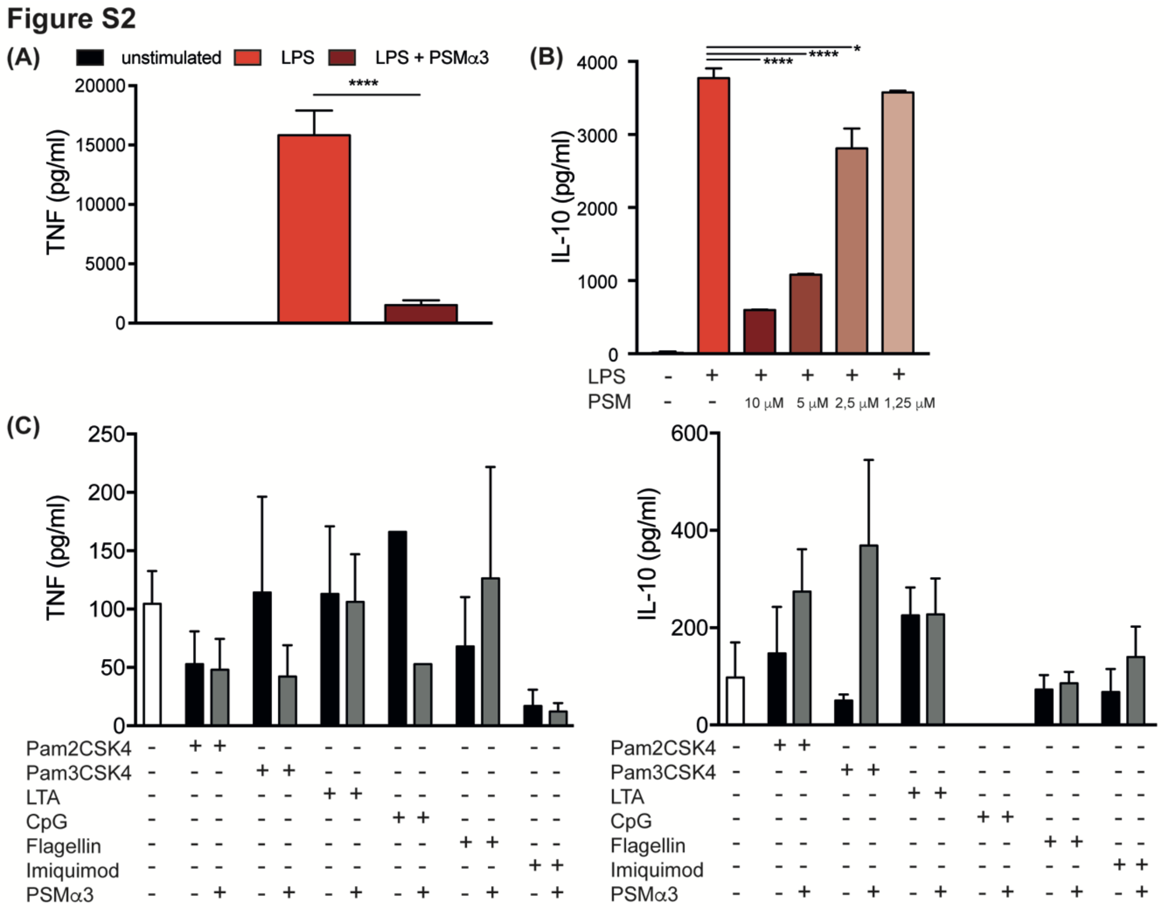

Figure S2: Cytokine production

(A) MoDCs were treated with LPS with or without PSMα3 for 24 h. The cell culture supernatants were collected and analyzed for TNF production by sandwich ELISA. (B) MoDCs were treated with LPS without or with the indicated concentrations of PSMα3 for 24 h. The cell culture supernatants were collected and analyzed for IL-10 production by sandwich ELISA. (C) MoDCs were treated with 100 ng/mL Pam2CSK4 (for TLR2/TLR6), 1 µg/mL Pam3CSK4 (for TLR1/TLR2), 1 µg/mL CpG ODN 2395 (for TLR9), 5 µg/mL Imiquimod (for TLR7), 2 µg/mL Flagellin (for TLR5) or 10 µg/mL LTA (for TLR2/TLR4) in combination with or without PSMα3 (10 µM). The cell culture supernatants were collected after 24 h and analyzed for TNF and IL-10 production by sandwich ELISA. *$p < 0.05$, or ****$p < 0.0001$, one-way ANOVA with Turkey's posttest or Kruskal-Wallis with Dunn's posttest.

Figure S3

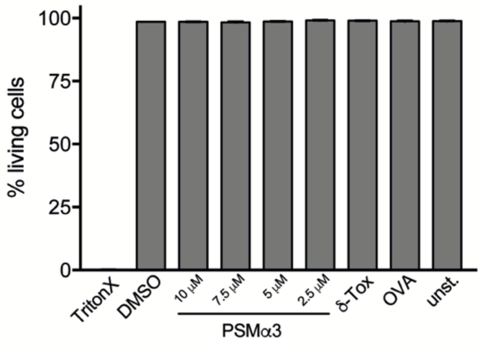

Figure S3: PSMα3 treatment has no effect on the viability of moDCs

MoDCs were either not treated (unst.) or incubated with 1% Triton X-100, 2% DMSO, PSMα3 (2.5 μM, 5 μM, 7.5 μM or 10 μM), 10 μM δ-Toxin or 5 μg/mL OVA for 10 min. The cell culture supernatants were used for LDH release assay (Fig. 3) and the cells were stained with 7-AAD and analyzed by flow cytometry for living cells. The graph shows the frequency of living cells (One representative experiment of n=3 independent experiments; mean ± SEM).

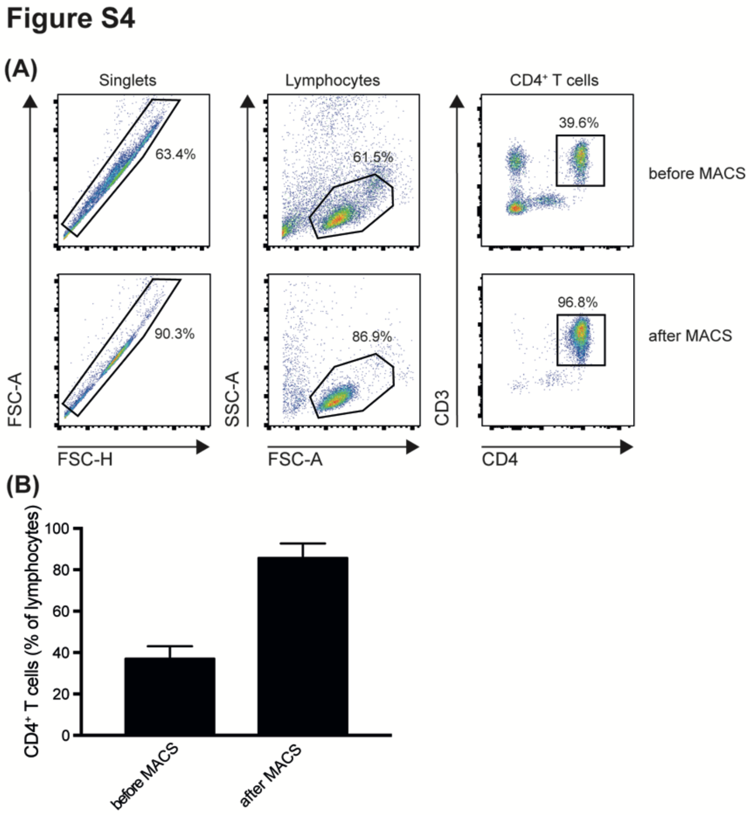

Figure S4: CD4$^+$ T cell purity

Human CD4$^+$ T cells were isolated by performing a magnetic bead assay and their purity was analyzed by flow cytometry. **(A)** The dot plots show the frequency of the CD3$^+$CD4$^+$ T cells before and after MACS. **(B)** The histogram shows the frequency of the CD3$^+$CD4$^+$ T cells before and after MACS with the MojoSort™ Human CD4 Naïve T Cell Isolation Kit (BioLegend) of collected data (n>5).

Figure S5

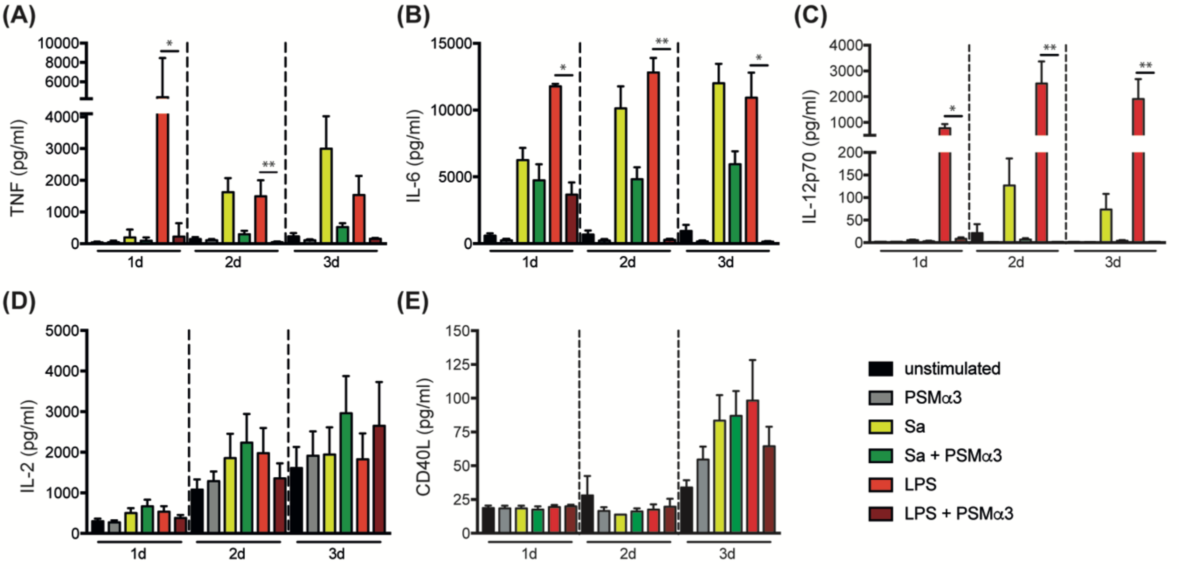

Figure S5: PSMα3 treatment of moDCs modulates cytokine production in co-culture with T cells

MoDCs treated for 24 h with Sa lysate or LPS with or without PSMα3 or PSMα3 alone were co-cultured with CFSE-labeled naïve $CD4^+$ T cells for 3 d. Cell culture supernatants were collected after 1 d, 2 d or 3 d and analyzed for TNF **(A)**, IL-6 **(B)**, IL-12p70 **(C)**, IL-2 **(D)** and CD40L **(E)** production (n≥3 performed in triplicates; mean ± SEM). *p<0.05, **p<0.005, ***p<0.001 or ****p<0.0001, one-way ANOVA with Turkey's posttest or Kruskal-Wallis with Dunn's posttest.

Figure S6

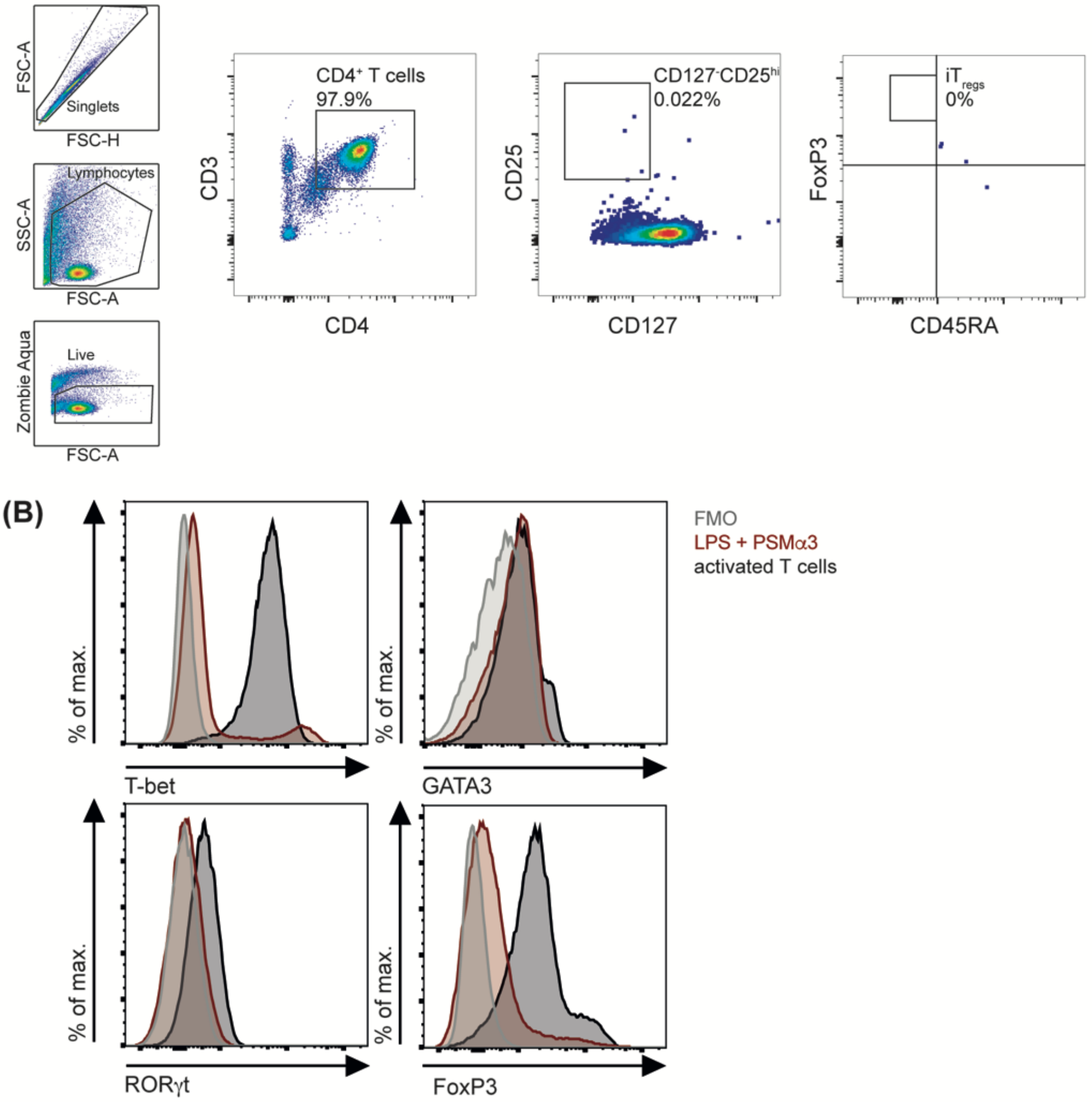

Figure S6: Flow cytometry controls

(A) Naïve $CD4^+$ T cells were treated with LPS and PSMα3 and cultured for 4 d without moDCs. iT_{regs} were analyzed by flow cytometry and characterized as $CD4^+CD127^-CD25^{hi}CD45RA^-Foxp3^{hi}$ cells. (B) Representative histogram overlays of intracellular staining for the transcription factors T-bet, GATA3, RORγt, and FoxP3. The grey peaks represent the fluorescence minus one controls (FMO), the red peaks show the staining moDCs treated with LPS and PSMα3 and the black peak shows the staining of T cells, which were activated with Dynabeads (Human T-Activator CD3/CD28 Proliferation) and cultured with 10 ng/ml IL-4 for 4 d (GATA-staining).

Figure S7

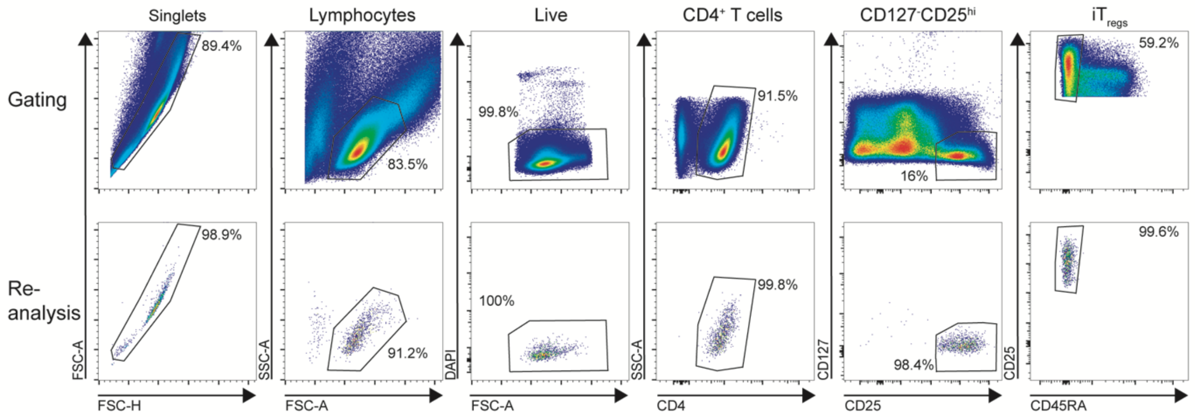

Figure S7: Gating strategy and purity of sorted iT$_{regs}$

MoDCs treated for 24 h with LPS and PSMα3 were co-cultured with naïve CD4$^+$ T cells for 4 d. CD4$^+$CD127$^-$CD25^{hi}CD45RA$^-$ iT$_{regs}$ were purified by FACS sorting. The dot plots show the gating strategy and the re-analysis of the sorted iT$_{regs}$.

Figure S8

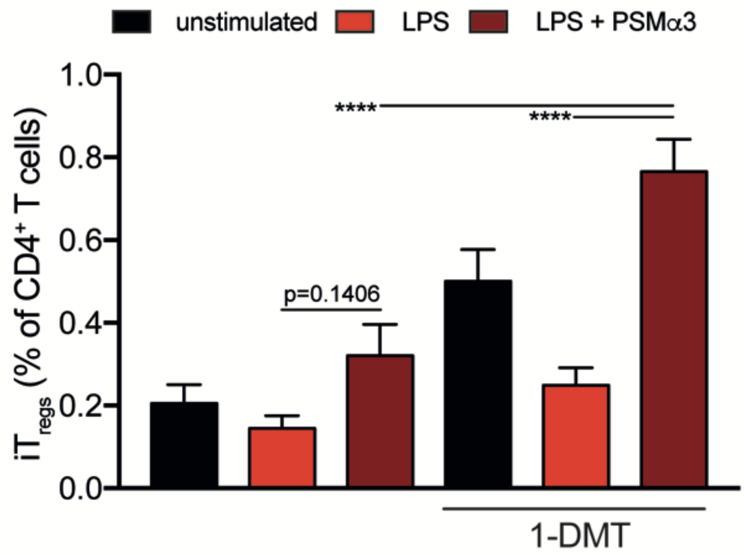

Figure S8: IDO inhibition does not prevent PSMα3-induced increase of iT$_{regs}$ frequency
MoDCs were treated or not with the IDO inhibitor 1DMT for 1 h prior to treatment with LPS in combination with PSMα3 for 24 h. Thereafter, DCs wer co-cultured with CFSE-labeled naïve CD4$^+$ T cells for 4 d. iT$_{regs}$ were analyzed by flow cytometry and characterized as CD4$^+$CD127$^-$CD25^{hi}CD45RA$^-$Foxp3^{hi} cells. The histograms show the frequency of iT$_{regs}$ from CD4$^+$ T cells. (n=4 performed in triplicates; mean ± SEM). ****$p < 0.0001$, one-way ANOVA with Turkey's posttest.

Figure S9

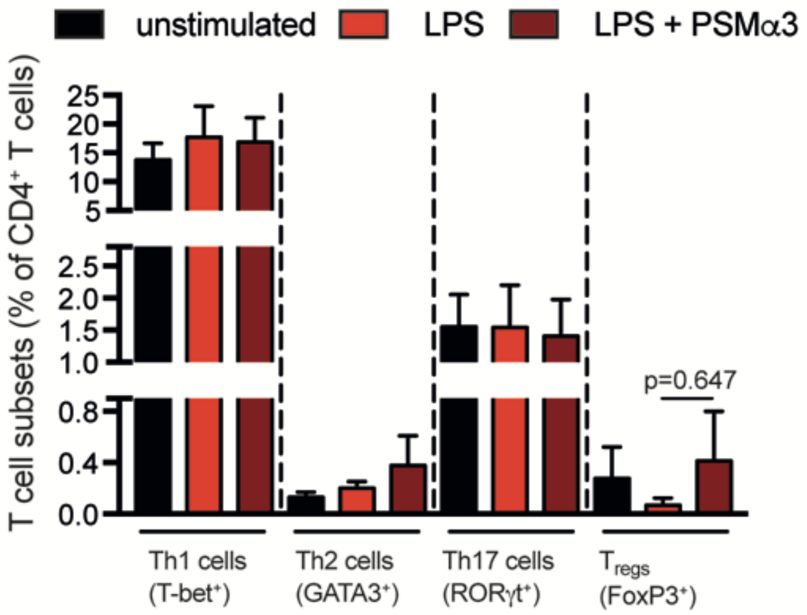

Figure S9: Autologous T-cell assay
MoDCs from spondyloarthritis patients were treated for 24 h with LPS with or without PSMα3 and co-cultured with CFSE-labeled $CD4^+$ T cells from the same patient for 4 days. The different T cell subsets were analyzed by flow cytometry. The graph shows the frequency of the $T\text{-bet}^+$ Th1, $GATA3^+$ Th2, $ROR\gamma t^+$ Th17 cells and $CD127^-CD25^{hi}CD45RA^-Foxp3^{hi}$ iT_{regs} from $CD4^+$ T cells (n=4 patients; mean ± SEM). One-way ANOVA with Turkey's posttest or Kruskal-Wallis with Dunn's posttest.
